# Radiation Therapy for Tracheobronchial Metastases from Head and Neck Squamous Cell Carcinoma

**DOI:** 10.7759/cureus.3151

**Published:** 2018-08-15

**Authors:** Nikhil Yegya-Raman, Sachin R Jhawar, Salma K Jabbour, Sabiha Hussain, Sung Kim

**Affiliations:** 1 Department of Radiation Oncology, Rutgers Cancer Institute of New Jersey, New Brunswick, USA; 2 Division of Pulmonary and Critical Care Medicine, Robert Wood Johnson University Hospital, New Brunswick, USA

**Keywords:** head and neck carcinoma, squamous cell carcinoma, tracheobronchial, metastases, radiation therapy, locoregional control

## Abstract

Tracheobronchial metastases from head and neck squamous cell carcinoma (HNSCC) represent a rare occurrence, with few reported cases in the literature. Here, we present two patients with HNSCC who developed tracheobronchial metastases at different time points in their disease course. Patient 1 presented with a localized tonsillar primary, underwent tonsillectomy and post-operative radiation therapy to the tumor bed and bilateral neck, and experienced multiple subsequent metastases in different locations throughout the tracheobronchial tree. Each time, she received surgery and/or chemoradiation therapy to the metastatic lesion(s). Patient 2 presented with a supraglottic primary metastatic to the carina and both mainstem bronchi, and, based on patient 1’s recurrence pattern and a suspicion for direct tumor extension, was treated upfront with definitive chemoradiation from the supraglottis down through much of the tracheobronchial tree. A year out from treatment, patient 2 has achieved excellent locoregional control with few treatment-related toxicities but unfortunately has developed new liver metastases not seen on pre-treatment imaging. This case highlights the difficulty in ascertaining the extent of metastatic spread for HNSCC patients with isolated tracheobronchial metastases and describes our approach to delivering curative-intent radiation therapy.

## Introduction

About 4%-26% of patients with head and neck squamous cell carcinoma (HNSCC) experience distant metastasis, most frequently to the lung, bone, and liver [1-2]. In contrast, tracheobronchial metastases are rare, with few reported cases [3-5]. Most secondary tracheobronchial tumors (86%) result from a thyroid, esophageal, or lung primary and are believed to arise through lymphatic spread, hematogenous spread, or direct extension from adjacent structures [6].

Given the rarity of tracheobronchial metastases from HNSCC, the mechanism of spread and role of radiation therapy remain unclear. Herein, we present two patients with HNSCC. The first patient presented with a localized disease, and received post-operative radiation to the tumor bed and bilateral neck; this recurred multiple times in different locations throughout the tracheobronchial tree. The second patient presented with synchronous metastases to the tracheobronchial tree and, based on our experiences with the first patient, was irradiated from the larynx down through much of the tracheobronchial tree.

## Case presentation

Patient 1 is a 65-year-old female with no smoking history and rare alcohol use who presented with a left tonsillar mass, confirmed as p16+ squamous cell carcinoma (SCC). She underwent left tonsillectomy via transoral robotic surgery and left neck dissection, revealing 2/21 involved lymph nodes (largest measuring 1.3 cm) without extracapsular extension. Lymphovascular invasion was indeterminate and perineural invasion was not identified. All deep and peripheral margins were free of invasive carcinoma. The closest margin from the left tonsil resection was 0.5 mm and was associated with a deep soft tissue margin on the lateral aspect. She was staged as pT2N2bM0.

**Figure 1:**
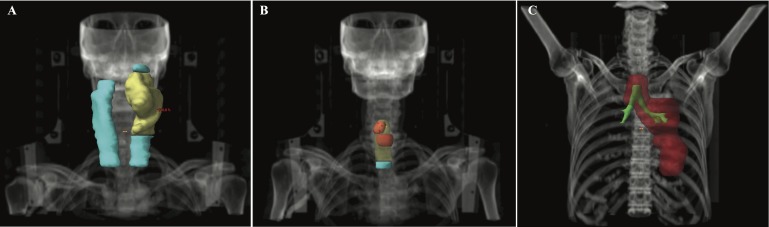
Radiation therapy target volumes for patient 1 (A) Initial treatment; yellow = planning target volume receiving 60 Gy (PTV60), cyan = PTV54 (B) Second recurrence; red = PTV66, yellow = PTV60, cyan = PTV54 (C) Third recurrence; red = PTV60, green = tracheobronchial tree

The patient received adjuvant radiation therapy of 60 Gy to the tumor bed and ipsilateral neck and 54 Gy to the contralateral neck (Figure 1A). Subsequently, she experienced four sequential recurrences, including three endotracheal and one lung. Each was visualized on positron emission tomography/computed tomography (PET/CT) and pathologically confirmed as p16+ SCC, strongly suggesting metastases from the original tonsillar primary. (1) Thirteen months after the initial treatment (completion of adjuvant radiation therapy), she experienced a 7 mm endotracheal metastasis just inferior to the level of the thyroid isthmus, for which 4 cm of the trachea was resected with negative margins. (2) Two years after the initial treatment, she experienced a 6 mm endotracheal metastasis just inferior to the cricoid cartilage on the left and a 3 mm endotracheal metastasis just inferior to the right vocal cord, for which she received 66 Gy (Figure 1B), concurrently with cetuximab, after an incomplete resection. She also received 60 Gy and 54 Gy to a small portion of the trachea extending inferiorly. (3) Two and a half years after the initial treatment, she developed a 16 mm metastatic focus in the left lower lobe of the lung and malignant left hilar adenopathy, for which she received 60 Gy via proton beam therapy (Figure 1C), concurrently with carboplatin and paclitaxel. (4) Lastly, three years after the initial treatment, she developed an endotracheal metastasis 15 mm below the left vocal cord. She was scheduled for another tracheal resection but was lost to follow-up at our institution.

Patient 2 is a 62-year-old male with a 75-pack year smoking history and rare alcohol use who presented asymptomatically with a left neck mass, confirmed as SCC on biopsy. PET/CT showed hypermetabolic activity in the right aryepiglottic fold without corresponding CT findings and multiple involved ipsilateral lymph nodes. Routine workup with triple endoscopy revealed the aryepiglottic fold lesion, as well as <2 mm lesions in the carina and right/left mainstem bronchi, which were not distinct on PET/CT. A biopsy of each of these lesions revealed SCC, which stained positive for p40 and negative for TTF-1, confirming the tumor’s supraglottic origin. Testing for p16 was not performed because of the non-oropharyngeal primary. The patient was staged as cT2N2bM1.

**Figure 2:**
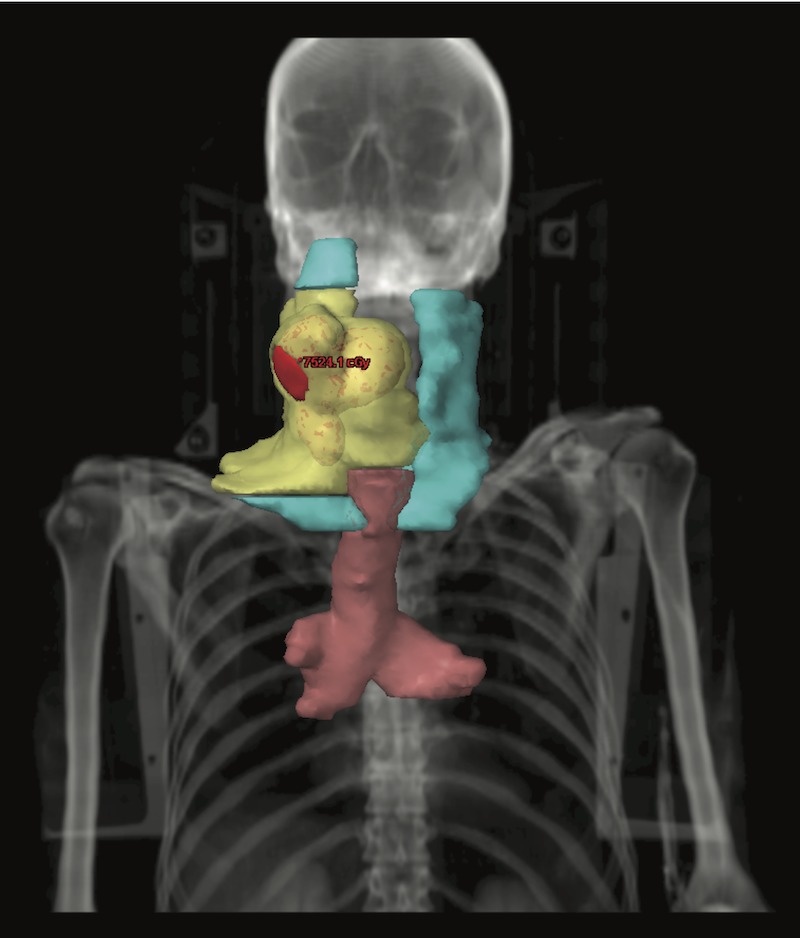
Radiation therapy target volumes for patient 2 Red = planning target volume receiving 70 Gy (PTV70), yellow = PTV60, cyan = PTV54, pink = PTV50

The patient received definitive radiation therapy concurrently with weekly cisplatin. Intensity-modulated radiation therapy (IMRT) was used to deliver a total of 70 Gy to the aryepiglottic fold primary lesion, 60 Gy to the ipsilateral neck and the remainder of the larynx, 54 Gy to the contralateral neck, and 50 Gy to most of the tracheobronchial tree (Figure 2). When treating the tracheobronchial tree, four-dimensional computed tomography (4DCT) simulation was used to define the internal target volume, which was expanded 0.5 cm volumetrically to form the clinical target volume and an additional 0.5 cm to form the planning target volume. Dosimetric parameters to select organs at risk (OARs) are shown in Table 1.

**Table 1:** Dosimetric parameters to organs at risk for patient 2 Vx, volume receiving ≥x Gy

Parameter	Value
Spinal cord	
Max dose (Gy)	34.3
Esophagus	
Mean dose (Gy)	30.7
V50 (%)	40.4
Lung	
Mean dose (Gy)	13.5
V20 (%)	19.6

During treatment, the patient experienced grade 3 esophagitis, resulting in a 14 lb weight loss and requiring a feeding tube. Additionally, he was hospitalized with aspiration pneumonia. Nevertheless, he completed his radiation therapy without breaks.

Three months after chemoradiation therapy completion, PET/CT showed partial to complete resolution of hypermetabolic activity at the right supraglottic region, as well as partial to complete resolution of malignant cervical lymphadenopathy. He tolerated food exclusively by mouth. Ten months after the completion of therapy, the patient remained free of other treatment-related toxicities. Direct laryngoscopy at the time showed some thickening of the right aryepiglottic fold, but no concerning lesions, and bronchoscopy showed a resolution of the previously documented lesions in the carina and mainstem bronchi. Unfortunately, soon thereafter, the patient presented with abdominal pain and was found to have histologically confirmed liver metastases not previously seen on imaging. At his most recent follow-up visit 12 months after treatment completion, the supraglottis showed no evidence of disease.

Table 2 summarizes and compares key clinical characteristics for patient 1 and patient 2.

**Table 2:** Comparison between patients 1 and 2

	Patient 1	Patient 2
Location of primary tumor	Left tonsil	Right aryepiglottic fold
Risk factors	Human papillomavirus; no known work/environmental exposures	Tobacco; no known work/environmental exposures
Treatment to primary	Left tonsillectomy and left neck dissection, followed by adjuvant radiation therapy (60 Gy to the tumor bed and ipsilateral neck and 54 Gy to contralateral neck)	Definitive concurrent chemoradiation therapy (cisplatin; 70 Gy to right aryepiglottic fold, 60 Gy to the ipsilateral neck, and 54 Gy to contralateral neck)
Timing of tracheobronchial metastases	Metachronous	Synchronous
Location and treatment of metastases	Endotracheal, inferior to the level of thyroid isthmus; resection of 4 cm of the trachea with negative margins Endotracheal, inferior to the level of cricoid cartilage on the left and right vocal cord; incomplete resection, then 66 Gy to residual disease concurrent with cetuximab Left lower lobe and left hilum; 60 Gy concurrent with carboplatin/paclitaxel Endotracheal, inferior to the left vocal cord; lost to follow-up	Carina, right and left mainstem bronchi; 50 Gy to much of the tracheobronchial tree (treated simultaneously with the primary tumor) Liver; started pembrolizumab
Disease-free interval after initial treatment	13 months until first tracheobronchial metastasis	11 months until liver metastasis; free of locoregional disease at 10 and 12-month follow-up

## Discussion

This report suggests that for HNSCC with tracheobronchial metastases, contiguous irradiation of both sites is feasible. Patient 1’s multiple recurrences within the tracheobronchial tree led us to treat patient 2’s synchronous metastases to the tracheobronchial tree more aggressively and to a larger radiation target volume. Patient 2 experienced few treatment-related toxicities and remains free of locoregional disease. However, he later developed liver metastases, which, admittedly, limits the interpretation of the efficacy of radiation therapy to the majority of the tracheobronchial tree.

Little is known about the role of definitive radiation therapy for tracheobronchial metastases. Isolated case series suggest that irradiation of an endotracheal metastasis from HNSCC is achievable; however, in these cases, metastases occurred years after the initial treatment of the primary lesion (not synchronously) and alongside lung metastases [4-5]. For primary tracheal tumors (of which ~45% are SCC), observational data indicates that radiation therapy may prolong survival [7] and Behringer et al. suggest chemoradiation therapy to 60-66 Gy as a reasonable option for inoperable patients [8].

Because patient 1 developed multiple lesions, each confirmed as p16+ SCC, in different locations throughout the tracheobronchial tree after focal treatment for her tonsillar primary, we presumed these were more likely to represent metastases than multiple primaries. It is possible that her airway was exposed to human papillomavirus (HPV), thereby leading to multiple primary malignancies by way of “field cancerization” [9]. However, Xu et al. found that among 406 patients with oropharyngeal SCC, non-smokers with HPV-related SCC (both of which apply to patient 1) had the lowest risk of developing second primary tumors in the upper aerodigestive tract [10]. Instead, we posit that perhaps her tracheobronchial tree was seeded at some point during intubation, which could account for the multiple intraluminal metastases in different locations. This mechanism of seeding of the tracheobronchial tree remains speculative; however, lymphatic or hematogenous spread resulting in numerous endotracheal lesions would be unusual. Seeding of HNSCC at tracheostomy [11], gastrostomy [12], and biopsy [13] sites have been described in the literature.

For patient 2, we also suspected that there may have been direct tumor extension to the tracheobronchial tree, given the multiple biopsy-proven metastases in close proximity within the carina and mainstem bronchi. We speculate that he may have aspirated a cluster of tumor cells from the aryepiglottic fold primary tumor. We decided to treat him more aggressively with the hope of achieving superior tumor control. The more typical approach may have been to include only the carinal and mainstem bronchi lesions in the radiation target volumes and to assume that chemotherapy alone would treat any micrometastases. However, such an approach proved unsuccessful for patient 1’s second recurrence. Given the possibility for other lesions in patient 2’s proximal tracheobronchial tree, we proceeded to treat the entire region after it was determined that this was dosimetrically feasible.

Expansion of the radiation target volumes to include the tracheobronchial tree is primarily limited by doses to OARs and toxicities. For patient 2, our decision to deliver 50 Gy instead of 60-66 Gy to the trachea and mainstem bronchi represented a balance between achieving control tumor control and minimizing doses to OARs. Esophagus and lung mean dose, lung volume receiving ≥20 Gy, and spinal cord maximum dose were all below the National Comprehensive Cancer Network’s recommended thresholds for conventionally fractionated therapy for non-small cell lung cancer. His main toxicity, grade 3 esophagitis, resolved completely by three months post-treatment. Still, careful monitoring for long-term toxicities, including tracheoesophageal fistulas, appears warranted. For patients receiving these sizable target volumes, it seems prudent to recommend IMRT or proton beam therapy, appropriate motion management techniques (e.g., 4DCT), and daily image guidance to improve the therapeutic ratio.

Another important point is that patient 2’s tracheobronchial lesions did not appear on PET/CT, only on bronchoscopy performed as part of triple endoscopy. The utility of routine triple endoscopy for HNSCC remains uncertain, perhaps as a result of the increasing use of PET/CT. However, as clinicians rely more on PET/CT and less on triple endoscopy, it is possible there will be more undetected second primary malignancies or micrometastases into the airway, given the lower spatial resolution of PET/CT. Guardiola et al. found that among 487 patients with HNSCC and no evidence of metastasis on CT scans of the thorax and head/neck, triple endoscopy detected second synchronous primary invasive carcinomas of the lung and esophagus in 1% (n=5) and 2% (n=10) of patients, respectively. Those with SCC of the hypopharynx carried the highest risk of esophageal carcinoma. Of note, this study did not incorporate PET/CT or assess the impact of these second malignancies on survival [14].

## Conclusions

This report describes our experience managing two patients with HNSCC who developed tracheobronchial metastases at different points in their disease course. Because patient 1 recurred multiple times throughout the tracheobronchial tree despite local therapy to the metastatic lesions, we decided to treat patient 2’s synchronous metastases more aggressively to a larger radiation target volume, which did lead to locoregional disease control. In both cases, we speculated the tracheobronchial metastases may have arisen through direct extension. However, patient 2 eventually developed liver metastases, which limits the interpretation of our approach to delivering curative-intent radiotherapy to much of the tracheobronchial tree. In light of this outcome and considering the relatively short follow-up for patient 2, our intention is primarily to report our experience treating these patients. Given the few reported cases of HNSCC with tracheobronchial metastases in the literature, we hope our experience can be of value to other clinicians who may face this rare situation.
